# Optimised electronic patient records to improve clinical monitoring of people living with HIV (MONART trial) intervention: a qualitative process evaluation

**DOI:** 10.1186/s12913-026-14827-1

**Published:** 2026-05-30

**Authors:** Rujeko Samanthia Chimukuche, Sadiyya Sheik, Zandile Mthethwa, Thabisile Mjilo, Samke Nxumalo, Londiwe Nzimande, Nothando Ngwenya, Collins Iwuji

**Affiliations:** 1 Africa Health Research Institute, Durban, KwaZulu-Natal, South Africa; 2 Division of Infection & Immunity, University College London, London, WC1E 6BT UK; 3 School of Nursing and Public Health, University of KwaZulu-Natal, Durban, KwaZulu-Natal, South Africa; 4 Department of Global Health Infection, Brighton and Sussex Medical School, University of Sussex and University of Brighton, Falmer, UK; 5 Department of International Public Health, Liverpool School of Tropical Medicine, Liverpool, UK

**Keywords:** Process evaluation, Health interventions, CFIR and NPT frameworks, Viral load monitoring

## Abstract

**Background:**

Viral load monitoring systems enable early detection of antiretroviral therapy failure, allowing for timely interventions. Systemic gaps and infrequent viral load monitoring contribute to delayed identification of antiretroviral treatment failure, increased risk of drug resistance, and poorer health outcomes for people living with HIV.

**Methods:**

We conducted qualitative research to assess the delivery and quality of the MONART Trial intervention, which utilised a VL champion model comprising an upgraded TIER.net platform and a quality improvement process. The upgraded TIER.net platform involved technological enhancements to the public electronic ART database. We conducted fourteen in-depth interviews with viral load champions to evaluate competency and experience, sixteen interviews with patients to understand their user experiences and acceptability of the intervention. The analysis was guided by the Consolidated Framework for Implementation Research (CFIR) and the Normalisation Process Theory (NPT) to understand the delivery and quality of implementation.

**Results:**

Our findings showed that ongoing support and continuous training on the upgraded TIER.net systems enabled healthcare workers to better engage and understand the electronic ART database which freed up time. Healthcare workers reported that the upgraded TIER.net reduced workloads while patients’ acknowledged improvements in service delivery, notably the reduced waiting times at public health facilities.

**Conclusion:**

Our study highlighted both the structural and behavioural dimensions of implementing the MONART intervention. The analysis enabled us to better understand the factors influencing the implementation process. Our findings underscored the importance of tailored training and patient-centred approaches for the effective integration of interventions within embedded health systems.

**Table:** 

Contributions to the literature
The study demonstrates that successfully embedding interventions within routine health systems requires tailored, patient-centred implementation approaches. These findings contribute to the design of future interventions.
Our analysis integrates the CFIR and NPT frameworks, moving beyond simply identifying barriers and facilitators and instead providing an explanatory account of how and why implementation fails or succeeds. CFIR characterises the contextual conditions influencing implementation while NPT explains the mechanisms through which practices are performed, embedded, and sustained. This contributes to the literature by illustrating how contextual determinants and behavioural processes interact to shape implementation outcomes.

## Background

Globally, an estimated 40.8 million people were living with HIV (PLHIV) in 2024, of whom 31.6 million were accessing antiretroviral therapy [1]. Due to the broadened criteria for initiating ART, treatment is offered to everyone with a positive test result [2]. However, inconsistent adherence may result in virological failure and the emergence of drug resistance [3]. The potential spread of drug-resistant viruses is influenced by ART coverage, the length of the ART programme, and the proportion and total number of individuals experiencing virological failure [4–6] Factors contributing to the transmission of resistant viruses include the percentage of treatment failures with resistant strains, time spent on ineffective regimens, viral load (VL) in patients with resistant viruses, the fitness of the resistant virus, and the likelihood of transmission compared to those who are ART-naïve [4]. In the global north, efficient viral load monitoring (VLM) systems allow for early detection of treatment failures, enabling timely changes to alternative suppressive ART. However, in low-and-middle income countries, poor monitoring of individuals on ART, inconsistent viral load monitoring, suboptimal adherence, and poor retention in care all contribute to poor treatment outcomes.

As of 2024, South Africa has the largest HIV programme globally with 6.33 million individuals living with HIV receiving ART [1]. However, formative research utilising an electronic database of a programmatic ART cohort in rural KwaZulu-Natal has demonstrated infrequent viral load monitoring and sub-optimal management of virological failure [7]. Using participatory methods with several stakeholders, the MONART trial was developed, which proposed a combination of interventions (quality improvement and technological augmentation of the public electronic ART database) to address poor viral load monitoring and improve virological suppression.

### Trial setting

The trial was hosted by the Africa Health Research Institute (AHRI), an independent scientific research institute and a Wellcome Trust Africa and Asia Programme. The study was implemented in the Hlabisa subdistrict of uMkhanyakude district in northern KZN where AHRI operates a Health and Demographic Surveillance System (HDSS) since 2000 [8]. The HDSS is an extensive social, demographic, HIV and clinical research programme of an under-resourced rural population of 150,000 in an area of about 845 km². Hlabisa is at the epicentre of the HIV epidemic in South Africa, where HIV prevalence among men and women aged 25–44 years in 2019 was 39.9% and 62.4% respectively [8, 9]. It is situated in one of the poorest districts nationally; about 5% of adults have completed a higher education, 4% are covered by a medical aid scheme and unemployment rate is 62% [8]. Seventeen primary health care clinics provide HIV care within the subdistrict, fourteen of which participated in this research.

### MONART intervention

Interventions were co-designed with clinic staff, health implementers, policymakers, and PLHIV after reviewing a gap analysis to determine whether suboptimal VLM resulted from clients not being offered testing, test results not being filed, or missing data in TIER.net. Interventions are described in the study protocol [10]. Briefly, these comprised:

#### a)Quality improvement package

Building on the VL champion model [11], two trained nurse champions in each intervention clinic dedicated approximately 10 hours weekly to tracing clients with high VLs, recalling them for adherence counselling, repeat testing, or referral for second-line ART. All nursing and support staff received training on VL monitoring, ART guidelines, and the enhanced TIER.net dashboard developed for the trial.

#### b)Augmentation of TIER.net

TIER.net is an electronic register used in South African public healthcare facilities to manage HIV and TB patient data. It records clinical visits, treatment details and laboratory results such as viral load and CD4 count for each patient. Healthcare workers enter this information during patient visit to maintain an up-to-date record. The system supports tracking of patient outcomes and follow-up of those who missed appointments and can generate standardised reports for monitoring and evaluation at a facility, district, and provincial levels. These reports are used for clinical decisions and for reporting to the National Department of Health. The key limitation is that laboratory results are entered manually by data capturers from printed copies, which is time consuming and requires filing and sorting before capturing. This manual process increases the workload and can delay data availability for clinical decision making. To address this, the study aimed to enhance the software to enable daily automatic import of laboratory results into TIER.net.

Excel-based reports were imported into TIER.net using deterministic linkage rules combining patient identifiers (e.g., name, sex, birth date, lab, and folder numbers).

### Conceptual framework

**Fig. 1:**
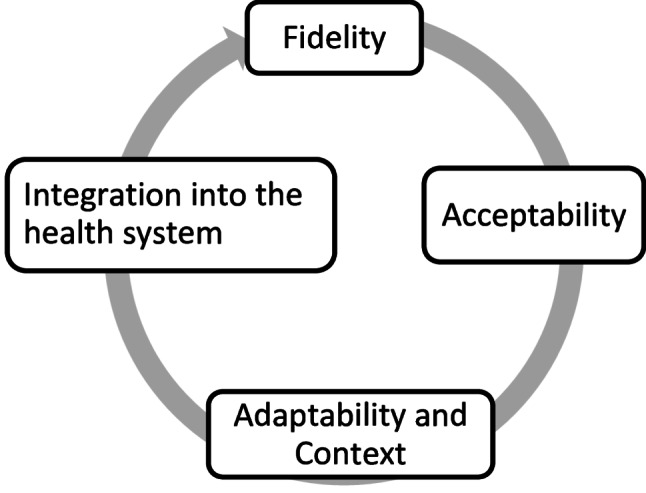
MONART process evaluation

We used the CFIR framework which includes five main domains (intervention characteristics, outer setting, inner setting, characteristics of individuals and process) that can inform implementation and evaluation criteria and can function as an explanatory framework in our analysis [12]. In our study we used select constructs of the CFIR domains to categorise the implementation process, explaining the outcomes [13, 14]. This enables the identification of explanatory themes that provide insight to the mechanisms within the implementation process. Normalisation Process Theory (NPT) has been used to understand challenges and barriers to the implementation of technology within healthcare settings. NPT as a mid-range sociological theory has four domains (coherence, cognitive participation, collective action, and reflexive monitoring) [15]. To complement the use of CFIR, NPT was used to inform the data collection tool guides which focused on fidelity, acceptability, adaptability, and integration (Fig. 1). The process evaluation aimed to investigate:

i. Self-efficacy to assess competency and experience of health care professionals to carry out and operate the upgraded TIER.net platform.
ii. The extent to which the QIP intervention was implemented and is consistent with the protocol and the extent to which training was provided as planned.
iii. The factors that influenced implementation of the QIP intervention and how the QIP and VLM intervention was integrated into health system practice.

## Methods

We conducted a process evaluation as a qualitative sub-study to assess the delivery and quality of the implementation in this setting. Fourteen in-depth interviews were conducted first with VL champions to evaluate competency and experience in using the upgraded Tier.Net across both intervention and control arms, focusing on understanding intervention delivery and its integration in healthcare settings. Sixteen interviews were conducted with patients to understand their user experiences at the intervention clinics and acceptability.

To guide the analysis, we applied the CFIR and NPT to examine both the determinants of implementation and their contribution to the process of shaping adoption of the upgraded TIER.net platform and the quality improvement (VL champion model). We used the same frameworks to assess patient experiences. We mapped the data according to the constructs of each framework (Table 1), with NPT informing analysis of how the intervention was operationalised in practice.

NPT focused on the process to illuminate how the healthcare providers implement and sustain the upgraded TIER.net platform in their routine practice and how the patients as recipients reflected on their experiences of the intervention. This included exploring how healthcare workers (HCWs) made sense of the upgraded TIER.net platform, operationalised it and how patients assessed and judged the effectiveness of this intervention within their setting.

**Table 1:** Mapping of the CFIR and the NPT frameworks for MONART process evaluation

Focus of interview guide	CFIR Domain	NPT Domain
**Patient interviews**		
Patients’ satisfaction with intervention	Outer setting and Process	Reflexive monitoring
Communication of intervention	Intervention characteristics	Reflexive monitoring (Individual appraisal)
Barriers affecting understanding of intervention	Outer setting (patient needs and resources)	Coherence
Overall experience of patient	Inner setting (culture)	Reflexive monitoring
**HCW interviews**		
Role and involvement of HCW	Process (engaging)	Cognitive participation
Important key factors in implementation of intervention	Inner setting (culture, implementation climate)	Coherence and Collective action
Adaptation needs	Intervention characteristics (adaptability)	Reflexive monitoring
External factors that impact on adaptation	Outer setting	Coherence
Other stakeholders’ feedback that contribute to adaptation	Process (reflecting and evaluating)	Reflexive monitoring
Challenges experienced	Process (executing)	Collective action and Reflexive monitoring

For healthcare workers, interview questions were focused on their roles, involvement in delivering the upgraded TIER.net platform and the quality improvement process of the VL champion model, as well as key implementation factors, adaptation needs, and external influences. For patients, the questions were focused on satisfaction, communication, barriers to understanding, and overall experience. The topic guide highlighted how both the QIP and VLM interventions were integrated into routine health system practice aligning with CFIR domains like process, inner and outer settings, and adaptability, and NPT domains including cognitive participation, coherence, collective action, and reflexive monitoring.

Three experienced social science researchers conducted the fourteen in-depth interviews (IDIs) with healthcare workers and sixteen face to face interviews with patients at the public health clinics in IsiZulu (the main local language). Table 2 gives a description of the study participants.

The fourteen VL champions were purposively recruited by nurse managers at their respective clinics, with one VL champion selected per clinic. The nurse manager serves as the head of each facility. The VL champions are trained to provide HIV treatment and care for PLHIV. Our patients were randomly selected using patient lists that were provided at the clinics.

Each interview lasted between 45 and 60 minutes. Participants were briefed on the purpose of the study and the researchers verified participants’ understanding of the study before obtaining informed consent. Participants reviewed the participant information documentation prior to giving their written informed consent to be involved. All interviews were digitally recorded, transcribed, and translated into English. Debriefing meetings were conducted after each interview with the interviewers and the lead author of this paper to improve probing, provide clarity on emerging themes and refine the topic guide where necessary.

**Table 2:** Study participants

Study Participants	Gender	Age range	Description	Arm
Health Care Workers	Fourteen females and two males	Not specified	13 Professional nurses 1 Operational manager working in public health care facilities	Seven intervention, seven control
Patients	Ten females and six males	30–76 years	Individuals attending public health care facilities for HIV care	Intervention

### Data analysis and interpretation

Data analysis was done manually led by the first author (RSC), with preliminary coding done by the second author (SS), reviewing of the analysis was further conducted by (NN). Further interpretation of the data was done by the research assistants who collected the data (ZM, TM and SN). First, team members independently read four transcripts to familiarise themselves with the data. A priori, a codebook was developed deductively using CFIR and NPT constructs and data were systematically coded against these categories.

While the analysis was primarily deductive, where relevant, additional codes were generated inductively to capture insights not fully encompassed by the predefined constructs. Where there was an overlap between CFIR and NPT constructs, codes were assigned based on the primary analytic focus of the data segment as CFIR was used mainly to capture contextual determinants of implementation and NPT was used to capture the behavioural processes through which the intervention was enacted in practice. To increase reliability and validity, the main codes were reviewed and discussed. Related deductive and inductive codes were compared across participant groups and organised into broader analytic categories. Through iterative discussion and refinement these categories were synthesised into three final themes: engagement and understanding, implementation, integration, adaptation, and context.

In this study, we recognised that our roles as researchers affiliated with the implementing institution with some involved in the broader MONART trial may have influenced the research process. The interviewers were not directly involved in the clinical care of patients however their affiliation to the trial through the institution may have influenced how participants responded. To minimise power dynamics, an attempt was made to ensure that the different roles of the researchers was communicated to participants so that they were aware that the interviewers were independent of routine service delivery and that all data would be anonymised and would not affect care or employment of the HCWs. When interpreting the findings, team members who collected the data helped provide context and deeper understanding of participants’ responses. At the same time, we were careful not to assume meaning beyond what participants shared. By working together and discussing different viewpoints, we aimed to balance our familiarity with the setting with a critical and reflexive approach to the data.

## Results

Findings are presented with explicit reference to relevant CFIR domains and NPT constructs to illustrate how contextual determinants and implementation processes shaped outcomes.

### Engagement and understanding of the upgraded TIER.net system

Our analysis guided by the CFIR (process, inner setting) and NPT (coherence, cognitive participation), showed that HCWs that were VL champions had limited engagement and understanding of the upgraded Tier.net and refrained from providing feedback. Limited understanding of the functionality of the system was evident among VL champions indicating limited coherence (NPT) and application as the champions reported minimal interaction with the system. VL champions shared that the system was predominantly used by data capturers mainly because they lacked the background training in health information systems.

*Well*,* I cannot comment much about Tier.net because I am not using it*,* the relevant person is the data capture*r Male VL champion. (Intervention arm).

During implementation of the upgraded TIER.net platform, **cognitive participation (NPT)** was shaped by team dynamics and roles as some HCWs expressed the importance of teamwork in the process and engaging with the upgraded system. They highlighted teamwork as an important factor within the **inner setting (CFIR)** to increase effectiveness.

*Ok my data capturers*,* [assisted with organizing and drawing the necessary files] this is because I work alone so*,* I do not have time really to draw files…yes.* Male VL champion (Intervention arm).

HCWs acknowledged the support they received from the project through various trainings and follow ups. This training and ongoing support strengthened **coherence (NPT)** and implementation **process (CFIR)** This helped them to be more effective in their work.

*We are given that opportunity*,* especially during trainings*,* even during their (MONART facilitator) clinic visits*,* they provide that opportunity for support…. we also discuss challenges like “you know*,* I have a problem with this*,* we are more than welcome to raise our issues* Female VL champion (Intervention arm).

Continuous support provided HCW and data capturers at the clinics with the opportunity to raise any implementation challenges and receive the necessary assistance enabling clearer understanding and more consistent engagement with the system.

*We started off with some confusion; there was just confusion until we went for the training. They first sent the guidelines without providing training for them*,* so it became clear that we were getting confused. There was a bit of misunderstanding because even if you follow them*,* there can still be misinterpretation. It’s better when there is training. After the training*,* everything became clear*,* it was much clearer…* Female VL champion (Intervention arm).

Adaptation needs such as continuous training improved significantly, resulting in a much clearer and more manageable process that is reflected among HCWs.

Patients highlighted a level of understanding of the upgraded TIER.net system which could be a good opportunity for integration. Such understanding demonstrated emerging **reflexive monitoring**. **(NPT)** as they reflected on the perceived benefits of the upgraded system such as potential increased efficiency and reduced waiting times at the clinic.

*Yes*,* according to me*,* I do not think I have a problem with how things are going to be*,* and I think the changing from hard copy files model will be better than to come to the clinic and wait for the nurses to locate your file. Yes*,* that is the other thing that makes the process to delay when coming to the clinic*,* the confirming of personal information like date of birth*,* maybe things will be faster if that can be changed.* Male,35 years.

Engagement and understanding highlighted how both patients and HCWs understood the upgraded TIER.net system. This includes how the intervention was communicated to patients and how individuals were willing and able to engage with it. It also reflects HCWs’ self efficacy and competence to deliver the intervention effectively.

### Implementation and integration of the upgraded TIER.net system (CFIR Domain- intervention characteristics and NPT collective action)

Participants highlighted that both intervention characteristics (upgraded TIER.net and VL champion) were helpful in enhancing their understanding of managing clients with a high viral load, outlining specific steps and counselling techniques. VL champions stated that the **intervention characteristics (CFIR)** particularly the dashboard functionality of the upgraded TIER.net enabled better management of patient care aspects that were previously overlooked, such as efficient tracking of viral loads. This supported **collective action (NPT)** by facilitating routine clinical tasks making it easier to identify unsuppressed patients and track the percentage of actioned cases which was particularly beneficial compared to earlier versions of Tier.net.

*It is very much relevant*,* I will not lie eh… as I have said that at first*,* we did not have something to measure the level of viral load*,* like for instance*,* Tier.net tells you the percentage of patients with viral load not actioned. You have this percentage of patients that are suppressed so*,* for a person who does not like counting*,* in fact it shows the percentage*,* and you will know yourself so*,* I think is very much relevant and it makes you not to relax by ignoring it.* Female VL champion (Intervention arm).

VL champions also stated that viral load measurements were now easier to track and the files could now be located a lot quicker. This new structured workflow in a way further strengthened **collective action (NPT)** as the intervention was embedded into routine practice and thus shifting from reactive care to systematic follow-up:

*It is another way that made things easier because at first the results would be sent and just sit; we would only notice when the person came in for their scheduled visit or when the results were captured in the file. They (nurses) would only start to act when the person was at the clinic*,* when you open the file and notice*,* ‘Wow! You had a high viral load in July; but now we have these results for action that are required and that we also need to correct on Tier (Tier.net). We have a system that says we should call these people with results for action. We open calls on Tier and close the call once we have found and dealt with the person. So now I think it is a bit better.* Male VL champion (Intervention arm).

Further, the HCWs stated that having a dedicated individual responsible for overseeing these results, ensures timely action. This also improves accountability within the **inner setting (CFIR)** and thus supporting coordinated action. *“So now if we get viral load results from the laboratory*,* we sit down and give ourselves time to action it. But now we have dedicated someone who is responsible for viral load results*,* she oversees those who need to be fast tracked and we action those results.”* Female VL (Intervention arm).

Patients that require urgent attention were identified timeously and this change has enhanced efficiency and effectiveness.

Patients reflected some appreciation of the communication sent to them by the clinics rather than waiting long hours during the implementation process.

*Eh*,* because going to sit at the clinic and wait in line takes a lot of time; waiting in the queue to get your file and do your vital signs becomes a long story (meaning it becomes a lengthy process). It’s much easier if you just receive messages while sitting at home and can continue with your work.* Female, 53 years.

Using the NPT construct of **reflexive monitoring**, results showed how the upgraded TIER.net system fits into the existing health system practices and reduced workloads and waiting times at the health facilities.

### Adaptation and contextual influences on implementation

HCWs interviewed acknowledged that the intervention led them to prioritize activities they had previously overlooked. The upgraded system improved awareness of viral load monitoring activities reflecting enhanced **coherence (NPT)** as the HCWs developed a clearer understanding of their responsibilities, “…*It was something understandable and we should have been doing it all along…”* Female VL (Intervention arm).

Participants also realised that there were aspects of their job responsibilities that they were not doing, *“But being involved in the MONART study*,* we were able to do this thing… we were not doing line list (referring to the existing Tier.net Viral Load reports)”* Female HCW.

The upgraded TIER.net system and the viral champion model interventions assisted by showing them areas that could be improved.

Although the system was seen as positive, implementation was however in resource constrained environment of staff shortages and heavy workloads **(inner setting CFIR)**. These contextual constraints limited the capacity for consistent **collective action (NPT)** and contributed to data discrepancies between patient files and electronic records which created gaps in monitoring unsuppressed patients. VL champions expressed the value of strengthening data capturing and monitoring processes, potentially improving accountability and clinic performance.

*…because of the shortage of staff we end up feeling it heavy*,* but what MONART is trying to do is to help us*,* because there is a common problem where we will find out that those patients whose viral loads are unsuppressed*,* their capturing does not tally with what is written in their files*,* so you will find out that people do viral load tests but nothing is written on their files.* Male VL champion. (Intervention arm).

Integration of both the upgraded TIER.net system and the quality improvement process (the VL champion model) into routine clinic workflow within the health facilities was facilitated by changes to the file organisation and tracking system reflecting a positive **implementation climate (inner setting CFIR).** HCWs reported how this enabled them to track patient files easily and making their work more efficient.

*Yes*,* it was very relevant because sometimes we were struggling to get patients’ files and they would just come and give us the easy way of using numbers to take out files so that if we receive viral load results then we will be able to pick up defaulters there and there.* Female VL (Intervention arm).

The implementation environment had an impact on the public health system as illustrated by another VL champion.

*I saw the importance of the intervention [upgraded Tier.net system] because it enabled us to be able to draw blood from patients and get the results soon but before we used to draw out blood from patients and it was not easy for us to get results promptly and patients will wait for 3 months for results to be ready* Female, VL champion (Intervention arm).

Our results guided by the CFIR inner setting domain showed that by introducing the upgraded TIER.net system that used numerical values to organize files, it simplified the process of accessing records. This shows a positive implementation climate for change. This efficiency allowed HCWs to promptly identify and follow up with patients who had defaulted after receiving viral load results.

Our findings show that previously, results in the electronic medical records were reviewed only when patients came to the clinic. However, with the upgraded Tier.net system, healthcare workers can now actively track patients who have been lost to follow-up.

*But now we are able to do so*,* it was just that if we are trying to do it*,* we had so many defaulters whereas our aim was to attend to that person at that time and discover the reason for visiting the clinic*,* we were not aware of the other ones who did not come back…so joining Monart opened our eyes that we have so many defaulters*,* there are so many patients who are “lost to follow up” then even if we were doing but there were not that much that we were getting as we were calling them (lost to follow up patients) on our own.* Female, VL champion (Intervention arm).

Viral load champions at the clinics introduced an action workflow, as a result, the HCWs have been using the Tier system to locate lost to follow up patients.

## Discussion

Using the combined CFIR and NPT frameworks in our analysis, findings from this nested qualitative study revealed that the upgraded TIER.net and the quality improvement process of the VL champion model was delivered as planned in the health facilities. Both HCWs’ efficiency and patients’ understanding significantly influenced the quality of the implementation process, highlighting the importance of clear communication, adequate training, and responsiveness to patients needs.

HCWs had limited direct engagement with the upgraded Tier.net. The application was instead utilised mainly by data capturers due to a lack of background training in health information systems for HCWs. This is an important finding and should be considered when planning future interventions utilising digital tools. HCWs also noted initial challenges, including confusion due to the lack of initial training and guidelines. However, following comprehensive training sessions, clarity improved significantly, facilitating smoother implementation of the intervention as observed in other HCW interventions [16, 17]. Our findings show that ongoing training was important because it allowed continuous building of skills and adaptation of the new interventions. This has been observed in other studies as beneficial to ensure fidelity and increase staff knowledge of the intervention [18, 19].

Our findings revealed that the MONART intervention addressed practical challenges within the clinics, such as improving the efficiency of patient file retrieval through systematic data capturing methods. Viral load champions who are also healthcare workers were instrumental in the application of the intervention outlining its effectiveness in solving the workload burden, health system challenges and alleviating the long waiting times faced by patients at the clinics. By supporting staff, they introduced a healthcare model based on the intervention outcomes facilitating a shift from being reactive to proactive management of patient results. Using the intervention characteristics described in CFIR outer setting domain, the study was also able to highlight patients’ satisfaction and understanding and how closely linked the intervention was tailored to their needs [20]. This is important when planning any new intervention, considering the feasibility of adoption and implementation from the start of the planning process [21].

Our findings demonstrate that effective implementation requires support beyond the introduction of new interventions to strengthening the broader health systems. Health policies should prioritise timely access to viral load results, ongoing training and clear communication for healthcare workers, and the integration of new interventions within existing health systems. In addition, creating supportive environments through strong leadership and adequate resourcing is critical to ensure sustainable health interventions.

These findings also have important implications for policy, clinical practice, and future research in the context of digital health in sub-Saharan Africa. For policymakers, the results suggest that successful interventions and approaches should be adaptable and integrated within existing systems. In clinical practice, the findings highlight the importance of healthcare worker engagement and understanding to enable digital interventions to be meaningfully embedded into routine care. The study shows that reliable infrastructure, ongoing training, and organisational support are critical.

## Limitations

This study was conducted in a single rural district, which may limit the transferability of the findings to other settings. The findings are most applicable to contexts with similar health system structures, cultures, languages, and socio-economic contexts. Some domains within the analysis frameworks overlapped conceptually, which made it challenging to assign findings to distinct categories. Finally, participant responses may have been influenced by social desirability or recall bias, particularly in retrospective accounts of implementation experiences.

## Conclusion

The combined use of the CFIR and NPT frameworks highlighted both the structural and behavioral aspects of the implementation of the MONART interventions. CFIR domains showed the context and process related factors, while NPT offered insight into how individuals made sense of, engaged with the upgraded TIER.net platform and the quality improvement process (the VL champion model) in practice. This analysis was essential for understanding the outcomes and contextual factors that influenced the implementation process, allowing the evaluation to explore what worked and why. Our findings highlight the need for tailored training, patient-focused and adaptive strategies, and effective integration of interventions into routine health system practice.

## Data Availability

The datasets generated and/or analysed during the current study are not publicly available due to identifiable or sensitive information but are available from the corresponding author on reasonable request.

## Abbreviations

ART: Antiretroviral treatment
CFIR: Consolidated Framework for Implementation Science
HDSS: Health and demographic system
HIV: Human immune deficiency virus
NHLS: National Health Laboratory Service
NICD: National Institute of Communicable Diseases
NPT: Normalisation Process Theory
PLHIV: People Living with HIV
VL: Viral Load
VLM: Viral load Monitoring
